# Use of Expert Panels to Define the Reference Standard in Diagnostic Research: A Systematic Review of Published Methods and Reporting

**DOI:** 10.1371/journal.pmed.1001531

**Published:** 2013-10-15

**Authors:** Loes C. M. Bertens, Berna D. L. Broekhuizen, Christiana A. Naaktgeboren, Frans H. Rutten, Arno W. Hoes, Yvonne van Mourik, Karel G. M. Moons, Johannes B. Reitsma

**Affiliations:** Julius Centre for Health Sciences and Primary Care, University Medical Centre Utrecht, The Netherlands; University of Sydney, Australia

## Abstract

**Background:**

In diagnostic studies, a single and error-free test that can be used as the reference (gold) standard often does not exist. One solution is the use of panel diagnosis, i.e., a group of experts who assess the results from multiple tests to reach a final diagnosis in each patient. Although panel diagnosis, also known as consensus or expert diagnosis, is frequently used as the reference standard, guidance on preferred methodology is lacking. The aim of this study is to provide an overview of methods used in panel diagnoses and to provide initial guidance on the use and reporting of panel diagnosis as reference standard.

**Methods and Findings:**

PubMed was systematically searched for diagnostic studies applying a panel diagnosis as reference standard published up to May 31, 2012. We included diagnostic studies in which the final diagnosis was made by two or more persons based on results from multiple tests. General study characteristics and details of panel methodology were extracted. Eighty-one studies were included, of which most reported on psychiatry (37%) and cardiovascular (21%) diseases. Data extraction was hampered by incomplete reporting; one or more pieces of critical information about panel reference standard methodology was missing in 83% of studies. In most studies (75%), the panel consisted of three or fewer members. Panel members were blinded to the results of the index test results in 31% of studies. Reproducibility of the decision process was assessed in 17 (21%) studies. Reported details on panel constitution, information for diagnosis and methods of decision making varied considerably between studies.

**Conclusions:**

Methods of panel diagnosis varied substantially across studies and many aspects of the procedure were either unclear or not reported. On the basis of our review, we identified areas for improvement and developed a checklist and flow chart for initial guidance for researchers conducting and reporting of studies involving panel diagnosis.

*Please see later in the article for the Editors' Summary*

## Abstract 2

Loes C. M. Bertens and colleagues survey the published diagnostic research literature for use of expert panels to define the reference standard, characterize components and missing information, and recommend elements that should be reported in diagnostic studies.

*Please see later in the article for the Editors' Summary*

## Editors' Summary

**Background:**

Before any disease or condition can be treated, a correct diagnosis of the condition has to be made. Faced with a patient with medical problems and no diagnosis, a doctor will ask the patient about their symptoms and medical history and generally will examine the patient. On the basis of this questioning and examination, the clinician will form an initial impression of the possible conditions the patient may have, usually with a most likely diagnosis in mind. To support or reject the most likely diagnosis and to exclude the other possible diagnoses, the clinician will then order a series of tests and diagnostic procedures. These may include laboratory tests (such as the measurement of blood sugar levels), imaging procedures (such as an MRI scan), or functional tests (such as spirometry, which tests lung function). Finally, the clinician will use all the data s/he has collected to reach a firm diagnosis and will recommend a program of treatment or observation for the patient.

**Why Was This Study Done?:**

Researchers are continually looking for new, improved diagnostic tests and multivariable diagnostic models—combinations of tests and characteristics that point to a diagnosis. Diagnostic research, which assesses the accuracy of new tests and models, requires that each patient involved in a diagnostic study has a final correct diagnosis. Unfortunately, for most conditions, there is no single, error-free test that can be used as the reference (gold) standard for diagnosis. If an imperfect reference standard is used, errors in the final disease classification may bias the results of the diagnostic study and may lead to a new test being adopted that is actually less accurate than existing tests. One widely used solution to the lack of a reference standard is “panel diagnosis” in which two or more experts assess the results from multiple tests to reach a final diagnosis for each patient in a diagnostic study. However, there is currently no formal guidance available on the conduct and reporting of panel diagnosis. Here, the researchers undertake a systematic review (a study that uses predefined criteria to identify research on a given topic) to provide an overview of the methodology and reporting of panel diagnosis.

**What Did the Researchers Do and Find?:**

The researchers identified 81 published diagnostic studies that used panel diagnosis as a reference standard. 37% of these studies reported on psychiatric diseases, 21% reported on cardiovascular diseases, and 12% reported on respiratory diseases. Most of the studies (64%) were designed to assess the accuracy of one or more diagnostic test. Notably, one or more critical piece of information on methodology was missing in 83% of the studies. Specifically, information on the constitution of the panel was missing in a quarter of the studies and information on the decision-making process (whether, for example, a diagnosis was reached by discussion among panel members or by combining individual panel member's assessments) was incomplete in more than two-thirds of the studies. In three-quarters of the studies for which information was available, the panel consisted of only two or three members; different fields of expertise were represented in the panels in nearly two-thirds of the studies. In a third of the studies for which information was available, panel members made their diagnoses without access to the results of the test being assessed. Finally, the reproducibility of the decision-making process was assessed in a fifth of the studies.

**What Do These Findings Mean?:**

These findings indicate that the methodology of panel diagnosis varies substantially among diagnostic studies and that reporting of this methodology is often unclear or absent. Both the methodology and reporting of panel diagnosis could, therefore, be improved substantially. Based on their findings, the researchers provide a checklist and flow chart to help guide the conduct and reporting of studies involving panel diagnosis. For example, they suggest that, when designing a study that uses panel diagnosis as the reference standard, the number and background of panel members should be considered, and they provide a list of options that should be considered when planning the decision-making process. Although more research into each of the options identified by the researchers is needed, their recommendations provide a starting point for the development of formal guidelines on the methodology and reporting of panel diagnosis for use as a reference standard in diagnostic research.

**Additional Information:**

Please access these Web sites via the online version of this summary at http://dx.doi.org/10.1371/journal.pmed.1001531.

- Wikipedia has a page on medical diagnosis (note: Wikipedia is a free online encyclopedia that anyone can edit; available in several languages)
- The Equator Network is an international initiative that seeks to improve the reliability and value of medical research literature by promoting transparent and accurate reporting of research studies; its website includes information on a wide range of reporting guidelines, including the STAndards for the Reporting of Diagnostic accuracy studies (STARD), an initiative that aims to improve the accuracy and completeness of reporting of studies of diagnostic accuracy

## Introduction

Different types of diagnostic studies, e.g., studies assessing the diagnostic accuracy of a single test or developing a multivariable diagnostic model, all face the key challenge of obtaining the correct final diagnosis in each subject. A final diagnosis is necessary to calculate the accuracy measures of the diagnostic test(s) or model(s) under study. Ideally, a single reference test to classify the condition of interest is preferred. For most conditions, however, such a single and error-free test, also known as a reference or “gold” standard, is not available [1]. This is problematic, as errors in the final disease classification can seriously bias the results [1],[2].

One strategy to overcome the lack of a single, imperfect reference test is to use multiple pieces of information to improve classification of the presence or absence of the disease. Several methods for utilizing multiple test results exist. These include so-called composite reference standards in which a predefined rule is used to combine different test results into a reference standard (for example, the combination of culture and PCR for the detection of infectious diseases) [3]; latent class analysis, where the multiple test results are modeled as functions of the unknown (or latent) disease status (for example, in the evaluation of the clinical accuracy in tests for pertussis) [4],[5]; and a so-called panel diagnosis, in which a group of experts determine the final diagnosis in each patient on the basis of all available relevant patient data (for example, often used in studies on heart failure) [1],[6].

**Figure 1:**
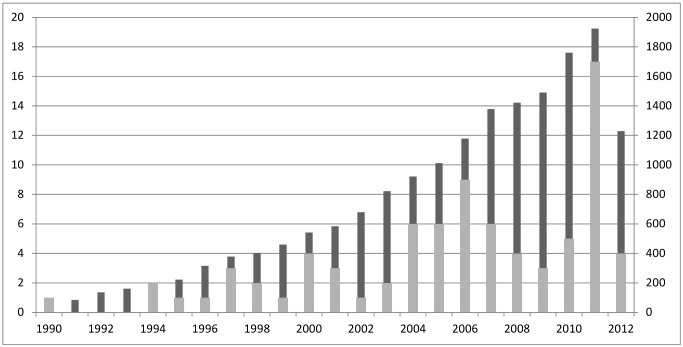
Distribution of search results over time. Dark grey columns represent the number of articles found with the search strategy, numbers displayed on right y-axis; light grey columns represent the articles included in the review after full text reading, numbers displayed on left y-axis.

In this review, we focus on panel diagnosis because its use appears to be increasing (Figure 1) and no formal guidance exists on the execution and reporting of this type of reference standard. Although terms like “consensus diagnosis” and “expert panel diagnosis” are also often used, we will use the more uniform term “panel diagnosis.” As a panel diagnosis largely resembles clinical practice in that multiple test results are assessed simultaneously by a clinician [7], it seems an acceptable method for obtaining a final diagnosis when a single gold standard test is lacking. Nonetheless, there are various ways to perform a panel diagnosis. These variations could arise from the chosen panel constitution and the methods applied to reach the decisions on the presence or absence of the target disease. Unfortunately, there is neither theoretical evidence, nor practical guidance on the preferred methodology to conduct panel diagnoses.

We performed a systematic review on reported panel diagnosis methodology to address the following aims: (1) To describe the variation in methods applied in published studies using a panel diagnosis; (2) To assess the quality of reporting of the methods related to the panel diagnosis process in these studies; (3) To provide initial guidance for researchers reporting an existing study or designing a new study involving a panel diagnosis.

## Methods

We performed our review in accordance to PRISMA guidelines for systematic reviews [8], but as methodological reviews differ from systematic reviews in several ways [9], not all items were applicable.

### Search and Inclusion Criteria

A PubMed search for articles on diagnostic studies using expert panels or consensus methods as final diagnosis was performed from its inception up to May 2012 by one of the authors (LCMB). The search strategy was explicitly very broad in order not to miss any relevant articles because of terminology used. The strategy included ([diagnosis] AND ([expert panel] OR [consensus methods] OR [consensus diagnosis])). The search was limited to studies in humans, and written in English. Because of theoretical saturation [9], meaning that additional searches will only add papers without adding information, we only performed the search in the largest electronic medical database (PubMed) and did not update the search beyond May 2012.

Studies had to meet three criteria to be included in the analysis: (1) The study was diagnostic, including studies on prevalence of the condition of interest, diagnostic accuracy, and multivariable (diagnostic) prediction models. (2) The reference standard used was based on the results of multiple tests, which were interpreted by multiple experts (two or more) to make a final diagnosis. (3) The study was an original report, excluding letters, editorials, case-reports, commentaries, and reviews.

### Data Extraction

Title and abstracts from the articles retrieved by the database search were screened and selected by LCMB for eligibility and identification for full-text reading. Articles were considered eligible for full-text reading when the abstract included clues that a panel diagnosis might have been used as reference standard. Full texts of the identified articles were read and the data-extraction form was completed by two observers in an independent (blinded) way (LCMB read and scored all articles and BDLB acted as the second reviewer in 120 articles and JBR in 64 articles).

The data extraction form (Protocol S1) was developed, piloted, and updated by LCMB, BDLB, and JBR and inspired by the STAndards for the Reporting of Diagnostic accuracy studies (STARD) guideline [10] and QUADAS-2 tool [11]. It was designed to collect descriptive information on how individual studies implemented the panel approach in their study and to collect normative information on the completeness of the reported methods (information levels A and B). General items about study aim(s), target disease(s), and reported reason(s) why a single reference standard was considered not appropriate were extracted. Detailed information on the methods used for panel diagnosis was also extracted, including: panel constitution, process of decision making, available tests results for the panel, blinding to the results of one of more tests, reproducibility of the panel diagnosis, and reported strengths and limitations of panel diagnosis. Discrepancies were resolved by discussion between the two reviewers. A formal level of agreement between the reviewers was not assessed. In only one paper agreement could not be reached between the two reviewers, and a third reviewer (JBR) was consulted.

## Results

### Search and General Study Characteristics

**Figure 2:**
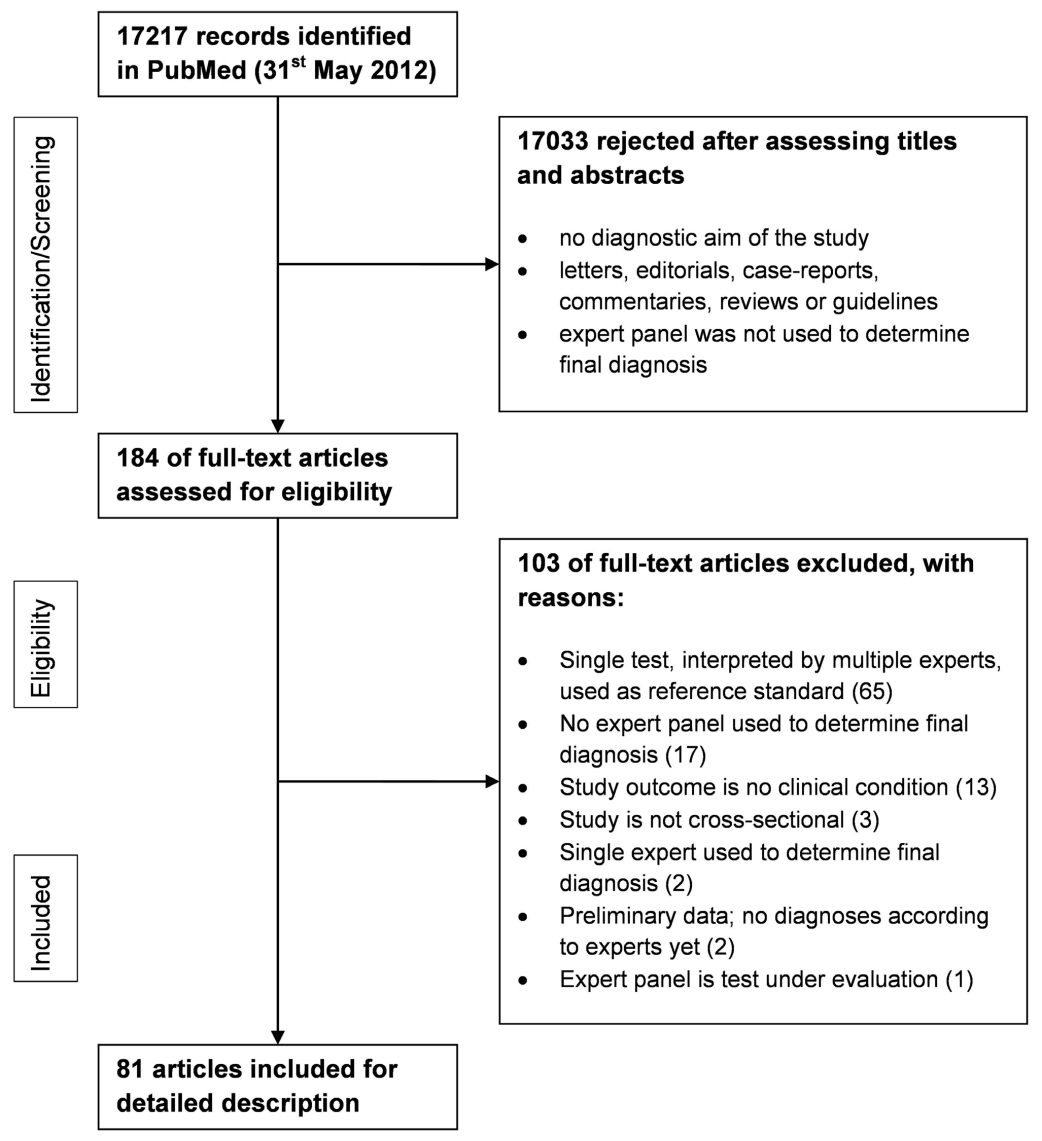
PRISMA flowchart of the selection of relevant papers.

The search yielded 17,217 potentially eligible articles on May 31, 2012. Applying the inclusion criteria to the abstracts reduced the number of papers to 184. Of these 184 articles, the full texts were retrieved and independently judged by two reviewers. Applying the inclusion criteria to the full texts resulted in 81 included articles to address objectives 1 and 2 (Figure 2). An overall quality assessment like QUADAS-2 [11] was not performed, but relevant items, such as if each patient received the final diagnosis in the same way, are included in the results.

Panel diagnosis was used in a broad spectrum of medical domains, but predominantly in the field of psychiatric disorders (30 of 81 papers, 37%), half of which pertained to dementia; cardiovascular diseases (17 papers, 21%); and respiratory disorders (ten papers, 12%). In seven studies (9%), the presence or absence of multiple diseases was assessed by the panel. Study characteristics are summarized in Tables 1–5 by medical domain: Table 1 for psychiatric disorders [12]–[41], Table 2 for cardiovascular disorders [42]–[58], Table 3 for respiratory disorders [59]–[68], Table 4 for studies with multiple target diseases [69]–[75], and Table 5 for diseases from other medical domains [76]–[92]. The median number of patients undergoing panel assessment of the included studies was 153 with a range of 12 to 4,474 patients.

**Table 1:** Study characteristics of articles assessing psychiatric disorders, *n* = 30.

Study Characteristics			Panel Members		Information for Panel Diagnosis			Decision Process					Validity	
Author, year	*n* Study Population	Study Aim	*n* Members	*n* Expertise	Available Information	Original Data Available	Blinding	*n* Disease Categories	*n* Evaluated by Panel	Initial Evaluation	Decision Making	Disagreements	Reproducibility	Comparison to Other Reference Test
Brugha, 2011 [15]	400	Accuracy	6	1	Q	?	N	4	400	Y	Consensus	?	Y	Y
Carnero-Pardo, 2011 [16]	139	Accuracy	2	1	PE, Q	?	Y	3	139	N	Consensus	Additional expert	?	N
Duberstein, 2011 [20]	191	Accuracy	?	?	PH, Q	?	?	?	191	?	Consensus	?	?	?
Girard, 2011 [23]	32	?	2	2	PH, I, Q	?	?	2	32	Y	Individual	?	?	?
Johnson, 2011 [27]	173	Accuracy	2	1	Q	?	?	2	173	?	Consensus	?	?	N
Ogunniyi, 2011 [32]	1,733	Prevalence	?	?	PE, BT, I, Q	?	?	3	1733	?	Consensus	?	Y	N
Plassman, 2011 [34]	217	Prevalence	?	4	PH, PE, BT, Q	?	N	2	217	?	Consensus	?	?	N
Hall, 2009 [26]	3,392	Prevalence	?	?	PH, PE, Q	?	Y	3	?	?	Consensus	?	?	?
Potter, 2009 [35]	645	Prediction model	?	2	PH, PE, Q	?	Y	2	645	?	Consensus	?	?	N
Steenland, 2008 [37]	204	Prediction model	2	1	PH, Q	?	Y	3	20	Y	Individual	Consensus	Y	?
Plassman, 2007 [33]	856	Prevalence	4	?	PH, PE, BT, Q	?	N	3	856	?	Consensus	Additional information	N	?
Baird, 2006 [12]	255	Prevalence	?	?	Q	?	?	?	255	?	?	?	Y	?
Graff-Radford, 2006 [24]	128	Accuracy	?	4	?	?	Y	?	128	?	Consensus	?	?	?
Sachdev, 2006 [36]	252	Prediction model	4	2	PH, PE, FT, I	?	?	?	252	?	Consensus	?	?	?
Boustani, 2005 [14]	227	Prevalence	4	4	PH, BT, FT, I	?	?	3	227	?	?	?	?	?
Williams, 2005 [41]	40	Accuracy	3	?	Q	?	?	2	40	?	Consensus	?	?	Y
De Koning, 2004 [18]	410	Accuracy	3	?	Q	?	Y	5	410	Y	Individual	Combined averages	?	?
Laurila, 2004 [28]	425	Accuracy	3	1	PH, I, Q	?	?	3	425	?	Consensus	?	?	?
Miller, 2001 [30]	56	Accuracy	3	?	PH, BT, I, Q	?	N	?	56	?	Consensus	?	?	?
Bienvenu, 2000 [13]	153	Prevalence	2	1	PH, PE, Q	Y	Y	4	153	Y	Individual	?	N	N
Magaziner, 2000 [29]	2,285	Prevalence	2	2	PH, Q	?	?	3	?	Y	Individual	Additional expert	Y	?
Weintraub, 2000 [39]	2,135	Prediction model	2	2	PH, Q	?	?	3	406	Y	Individual	Additional expert	Y	?
Fladby, 1999 [22]	40	Accuracy	?	?	?	?	?	2	40	?	Consensus	?	?	N
Ogunniyi, 1998 [31]	77	Prevalence	?	1	PH, BT, FT, I	?	?	?	77	?	Consensus	?	Y	?
Gulevich, 1997 [25]	185	Accuracy	3	3	PH, PE	Y	Y	3	185	Y	Consensus	?	?	?
Wiener, 1997 [40]	20	Inter-rater variability	2	1	Q	?	?	?	20	?	Consensus	?	?	?
Class, 1996 [17]	106	Prevalence	3	2	PH, PE, BT, FT, I	?	?	?	106	?	Consensus	?	Y	?
Tanenberg-Karant, 1995 [38]	196	Prevalence	?	1	PH, Q	?	Y	2	196	Y	Individual	Consensus	Y	?
Fennig, 1994 [21]	232	Accuracy	2	1	PH, Q	?	?	?	232	Y	Individual	Consensus	Y	?
Drake, 1990 [19]	75	Prevalence	?	?	PH, Q	?	N	2	?	Y	Consensus	Additional expert	?	?

Abbreviations: ?, not reported; BT, blood test; FT, function test; I, imaging; N, no; PE, physical examination; PH, patient history; Q, questionnaire. Y, yes;

**Table 2:** Study characteristics of articles assessing cardiovascular disease, *n* = 17.

Study Characteristics			Panel Members		Information for Panel Diagnosis			Decision Process					Validity	
Author, Year	*n* Study Population	Study Aim	*n* Members	*n* Expertise	Available Information	Original Data Available	Blinding	*n* Disease Categories	*n* Evaluated by Panel	Initial Evaluation	Decision Making	Disagreements	Reproducibility	Comparison to Other Reference Test
Assomull, 2011 [42]	120	Accuracy	3	1	PH, I	?	N	6	120	N	Consensus	Majority	?	Y
Doubal, 2011 [45]	355	Prediction model	3	3	BT, I, FU	Y	N	?	?	?	?	?	?	N
Kelder, 2011 [53]	47	Accuracy	3	3	PH, PE, BT, FT, I, FU	?	Y	?	47	?	?	?	?	?
Kelder, 2011 [52]	200	Accuracy	3	3	PH, PE, BT, FT, I, FU	?	Y	?	200	?	?	?	?	?
Oudejans, 2011 [56]	206	Prediction model	4	4	PH, PE, BT, I, FU	?	Y	2	206	N	Consensus	Considered absent	Y	N
Bosner, 2010 [43]	1,199	Prediction model	3	3	PH, PF, FT, FU	?	N	2	1199	?	?	?	?	?
Gaikwad, 2008 [46]	33	Accuracy	2	1	PH, I	?	?	2	33	N	Consensus	?	?	?
Hoffmann, 2007 [47]	70	Accuracy	2	1	PH, FT, I	Y	?	2	9	?	Consensus	?	?	?
Kantarci, 2007 [51]	33	Accuracy	2	2	I	Y	?	?	33	?	Consensus	?	?	?
Linn, 2007 [54]	19	Accuracy	3	?	PH, I, FU	N	?	?	19	?	Consensus	?	?	?
Nordenholz, 2007 [55]	254	Prevalence	2	1	I, DID	?	?	3	15	Y	Consensus	?	?	?
Hoffmann, 2006 [49]	103	Prediction model	2	2	PH, BT, FT, DID	?	Y	2	103	?	Consensus	Additional expert	?	Y
Hoffamnn, 2006 [50]	40	Accuracy	2	2	PH, BT, FT, DID	N	Y	2	40	?	?	Consensus	?	?
Hoffmann, 2006 [48]	100	Accuracy	2	1	MH, FT, I	Y	N	2	15	Y	Consensus	?	?	N
Trevelyan, 2003 [58]	401	Accuracy	3	2	PH, BT, FT	?	?	4	401	?	?	?	?	Y
Dao, 2001 [44]	250	Accuracy	2	1	PH, PE, BT, I, FU	?	Y	3	250	Y	Consensus	Additional information	?	?
Remy-Jardin, 2000 [57]	82	Accuracy	2	1	I	Y	?	2	82	?	Consensus	Additional information	?	?

Abbreviations: ?, not reported; BT, blood test; DID, discharge or preliminary diagnosis; FT, function test; FU, follow-up; I, imaging; N, no; PE, physical examination; PH, patient history; Y, yes.

**Table 3:** Study characteristics of articles assessing respiratory disorders, *n* = 10.

Study Characteristics			Panel Members		Information for Panel Diagnosis			Decision Process					Validity	
Author, Year	*n* Study Population	Study Aim	*n* Members	*n* Expertise	Available Information	Original Data Available	Blinding	*n* Disease Categories	*n* Evaluated by Panel	Initial Evaluation	Decision Making	Disagreements	Reproducibility	Comparison to Other Reference Test
Guder, 2012 [63]	405	Accuracy	2	2	PH, FT, I	?	N	2	405	?	Consensus	?	Y	?
Mohammed Hoessein, 2012 [64]	342	Accuracy	2	2	PH, PE, FT	?	N	2	342	N	Consensus	Additional expert	Y	?
Thieme, 2012 [68]	15	Accuracy	4	2	I	?	N	2	15	Y	Individual	Consensus	?	?
Broekhuizen, 2011 [60]	372	Accuracy	2	?	PH, PE, FT, FU	?	Y	2	372	N	Consensus	?	?	?
Broekhuizen, 2010 [59]	353	Prevalence	2	2	PH, PE, FT, FU	?	N	2	353	N	Consensus	Additional expert	Y	N
Szucs-Farkas, 2009 [67]	120	Accuracy	2	1	PH, I	?	N	2	120	?	Consensus	Additional expert	?	Y
Reinartz, 2006 [66]	53	Accuracy	?	?	BT, I, FU, DID	?	Y	?	53	?	Consensus	?	?	?
Chavannes, 2004 [61]	12	Accuracy	4	3	PH, PE, FT	?	Y	4	12	N	Consensus	?	?	N
Reinartz, 2004 [65]	83	Accuracy	?	?	BT, I, FU, DID	?	N	?	83	?	Consensus	?	?	?
Gauvin, 2003 [62]	30	Accuracy	3	?	PH. PE, BT, I	?	Y	2	30	Y	Individual	Consensus	?	?

Abbreviations: ?, not reported; BT, blood test; DID, discharge or preliminary diagnosis; FT, function test; FU, follow-up; I, imaging; N, no; PE, physical examination; PH, patient history; Y, yes.

**Table 4:** Study characteristics of articles assessing multiple diseases, *n* = 7.

Study Characteristics					Panel Members		Information for Panel Diagnosis			Decision Process					Validity	
Author, Year	*n* Study Population	Study Aim	Medical Domain(s)	*n* Target Disease	*n* Members	*n* Expertise	Available Information	Original Data Available	Blinding	*n* Disease Categories	*n* Evaluated by Panel	Initial Evaluation	Decision Making	Disagreements	Reproducibility	Comparison to Other Reference Test
Ray, 2006 [73]	514	Accuracy	CD, RD	8	2	6	PH, PE, BT, FT, I	N	N	?	514	Y	Individual	Additional expert	Y	?
Rutten, 2005 [74]	405	Prevalence	CD, RD	2	4	3	PH, PE, BT, FT, I	?	?	3	405	?	Consensus	?	?	?
White, 2005 [75]	69	Accuracy	CD, RD	6	3	3	PH, PE, I, FU, DID	?	N	2	69	?	Consensus	?	?	Y
Marshall, 2004 [71]	107	Accuracy	GD	3	6	3	PH, BT, I	N	N	2	107	N	Consensus	?	?	?
Jorgensen, 1998 [70]	148	Accuracy	CD, GD, MD, RD	6	7	3	PH, BT	?	Y	2	148	?	Consensus	?	?	Y
Geirnaerdt, 1997 [69]	78	Inter-rater variability	MD	2	2	1	PH, I	Y	Y	?	78	?	Consensus	?	?	Y
Martinez, 1994 [72]	50	?	CD, PD, RD	6	3	?	PH, PE, FT, FU	?	?	2	50	Y	Consensus	?	?	?

Abbreviations: ?, not reported; BT, blood test; CD, cardiovascular disorders; DID, discharge or preliminary diagnosis; FT, function test; FU, follow-up; GD, gastroenterological disorders; I, imaging; MD, musculoskeletal disorders; N, no; PD, psychiatric disorders; PE, physical examination; PH, patient history; RD, respiratory disorders; Y, yes;.

**Table 5:** Study characteristics of articles assessing diseases from other medical domains, *n* = 17.

Study Characteristics				Panel Members		Information for Panel Diagnosis			Decision Process					Validity	
Author, Year	*n* Study Population	Study Aim	Medical domain	*n* members	*n* Expertise	Available Information	Original Data Available	Blinding	*n* Disease Categories	*n* Evaluated by Panel	Initial Evaluation	Decision Making	Disagreements	Reproducibility	Comparison to Other Reference Test
Ham, 2012 [79]	127	Accuracy	DD	2	2	PH, PE, BT, I, FU	?	?	2	127	N	Consensus	?	?	?
Bisulli, 2011 [77]	101	Accuracy	ND	3	2	PH, I, Q, FU	?	Y	2	101	?	?	?	?	N
Gamez-Diaz, 2011 [78]	630	Accuracy	BD	3	3	PH, BT, I	?	Y	2	221	Y	Individual	Consensus	Y	Y
Van Randen, 2011 [90]	1,021	Accuracy	DD	3	2	PH, PE, BT, I, FU	?	N	?	1021	Y	Individual	Consensus	Y	?
Whiteley, 2011 [92]	356	Accuracy	ND	?	3	PH, PE, I, FU	?	Y	3	356	?	?	?	?	?
Hardie, 2010 [80]	51	Accuracy	DD	2	1	I	?	N	2	51	?	Consensus	?	?	N
O'Toole, 2010 [83]	75	Accuracy	MD	4	1	I	Y	Y	2	75	N	Consensus	Majority	?	N
Thabut, 2010 [89]	242	Accuracy	BD	3	?	PE, BT	N	?	3	242	Y	Individual	Consensus	?	?
Amour, 2008 [76]	276	Accuracy	ID	2	?	PH, PE, BT	?	Y	5	276	Y	Individual	Additional expert	Y	?
Humphries, 2008 [81]	44	Accuracy	UD	?	2	PE, I	?	?	?	3	?	Consensus	?	?	?
Lin, 2007 [82]	72	Accuracy	UD	2	1	PH, PE, I, FU	?	?	?	72	?	Consensus	?	?	?
Tadros, 2006 [87]	44	Accuracy	MD	?	?	I	N	N	2	44	?	Consensus	?	?	?
Otte, 2005 [84]	102	Accuracy	GD	?	?	PH, FT, I	?	N	?	102	?	Consensus	?	?	Y
Robin, 2005 [86]	261	Accuracy	ED	9	1	PH, FT	?	Y	4	261	Y	Individual	Consensus	?	?
Tepper, 2004 [88]	377	Prevalence	ND	4	?	PH	?	Y	?	377	?	?	Consensus	?	?
Penzkofer, 2002 [85]	80	Accuracy	ND	2	1	I	?	Y	2	80	?	?	?	?	?
Weih, 2001 [91]	4,744	Prevalence	ED	6	1	PH, PE, I	?	?	3	4744	Y	Individual	Consensus	?	N

Abbreviations: ?, not reported; BD, disorders of the blood; BT, blood test; DD, disorders of the digestive system; ED, eye disorders; FT, function test; FU, follow-up; GD, gastroenterological disorders; I, imaging; MD, musculoskeletal disorders; N, no; ND, disorders of the nervous system; PE, physical examination; PH, patient history; Q, questionnaire; UD, disorders of the genitourinary system; Y, yes.

The study aim of most papers (52 of 81 papers, 64%) was to assess the accuracy of one or more diagnostic tests. In 17 studies (21%) the aim was to determine the prevalence of a particular disease, and in seven studies the aim was to develop a multivariable diagnostic prediction model. In two articles (2%) the study aim remained unclear.

### Completeness of Reporting

Table 6 displays the proportion of articles that reported on different items related to panel constitution, information available for panel evaluation, and methods of decision making. Incomplete reporting was a common finding: information on panel constitution was missing in 20 (25%) studies, information on tests result presented to the panel was missing in 28 (35%) studies, and information about the decision process within the panel was incomplete in 56 (69%) studies. Overall, key information on panel methodology, related to STARD items [10] on the reference standard, was incomplete in 67 (83%) of the 81 included studies.

**Table 6:** The proportion of articles that reported on items related to panel constitution, information available and methods of decision making.

Item:	Number (%) of Articles
*Panel constitution*	
Number of panel members?	63 (78%)
Field(s) of expertise?	61 (75%)
*Information available for panel diagnosis*	
Which information was available for panel evaluation?	79 (98%)
Was original/raw data available?	10 (12%)
Blinding of tests to the panel?	53 (65%)
*Methods of decision making*	
Was the entire study population assessed by the panel?	71 (88%)
Disease classification? (e.g., present/absent)	58 (72%)
How were the decisions on disease status made?	71 (88%)
Handling of disagreements?	29 (36%)

Total number of studies is 81. The displayed items were inspired by the reporting guideline for diagnostic research. The number of articles represents those that reported something on the items concerning panel constitution, information available for panel diagnosis, and the methods of decision making. For example, 53 studies reported on blinding of tests to the panel; this could include listing the specific items that were not available for panel diagnosis (blinding) or the statement that all patient data and tests were available for panel diagnosis.

### Variation in Methodology across Studies

#### Panel constitution

Most panels used two members (29 of 63 papers, 46%), followed by three members (18 of 63 papers, 29%). The maximum reported number of members was nine. Different fields of expertise of the panel members were represented in the majority of studies (37 of 61 papers, 61%), with a maximum of six different fields of expertise.

#### Available information for panel diagnosis

Items from patient history and/or physical examination were used by the panel in 80% of the studies (63 out of 79 articles; two articles did not report on this item). Imaging results were also frequently used (43 of 79 articles, 54%). Blood tests, questionnaires, and function tests (such as spirometry) were each used for evaluation by the panel in 30% of studies (24 out of 79 studies). Information collected during follow-up was used by the panel in 21 studies (27% of 79 studies) and discharge or preliminary diagnoses of the treating physician were also presented to the panel in six studies.

#### Format of presentation to the panel

In 79 of the 81 articles, the available information was presented to the members as paper-based summaries. In nine (11%) of the 81 included studies, test results were also presented in their original (raw) form, such as original radiographic images.

In 32 papers (60% of 53 papers), panel members were blinded (i.e., results were withheld) to one or more test results. For most of these studies (23 of 32 studies), the members were blinded to the results of a specific index test under study. Two studies used staged unblinding of the test results, in which the diagnosis was assigned twice by the panel, first on all data but without the results of the index test and later including the index test results. The other 21 articles reported that all available patient data was included for panel diagnosis.

#### Decision-making process by the panel

The final diagnosis was determined only as “target disease present or absent” in the majority (33 of 58 studies; 57%) of studies. In the other 25 studies, multiple categories of estimated certainty for disease classification were used, with a maximum of six categories.

We observed many combinations of initial evaluation of the information by the panel members (individual or plenary), method of decision making by the panel, and how they handled disagreements across the panel members during the process of reaching a decision on the presence/absence of the target disease (Table 7). A plenary decision process was more frequently used than combining individual panel members' assessments into a majority decision (51 versus 17 studies).

**Table 7:** Observed combinations of the decision process used in the reviewed articles.

Initial Evaluation		Decision Process		Handling of Disagreements	
Type	*n*	Type	*n*	Type	*n*
Individual	24	Individual	17	Additional expert	4
				Discussion	10
				Other^a^	1
				Not reported	2
		Plenary	7	Additional information	1
				Additional expert	1
				Not reported	5
Plenary	11	Plenary	11	Additional information	1
				Additional expert	3
				Voting	2
				Not reported	5
Not reported	46	Plenary	34	Additional information	1
				Additional expert	2
				Discussion	1
				Not reported	30
		Not reported	12	Discussion	2
				Not reported	10

Initial evaluation of the information was done individually, during a plenary meeting, or no details were reported. Decisions on disease status were made by combining individual scores (individual), in a plenary meeting, or no details were reported. For Additional expert, another expert was consulted to resolve disagreements; for Discussion, disagreements were resolved through discussion with all members; for additional information, extra information was made available to members to resolve disagreements; for voting, disagreements are resolved by choosing the opinion of the majority.

a Averages of the panel members were calculated to decide on the disease status. For example, the panel members first assessed the information individually, decided on the diagnosis in a plenary meeting, and resolved disagreement by consulting an additional expert.

In 22 studies (31% of 71 articles), only a subgroup of patients was assessed by the entire panel. This subgroup often consisted of patients who were difficult to diagnose by individual assessment by the panel members (16 of these 22 studies). A pre-specified decision rule to select such subgroups of patients was applied in three papers; two studies used disagreement between multiple index-tests to identify the patients for panel assessment and another study defined subgroups for panel assessment on the basis of the information available per patient.

#### Validity of panel diagnosis

Twenty-seven papers reported the reproducibility of the panel diagnosis in their study. Kappa statistics or agreement percentages were reported in 17 articles (21% of 81 articles), of which seven studies evaluated the plenary decision process and ten studies reported the reproducibility of the individual assessments.

In addition to the panel diagnosis, ten studies (12% of 81 studies) also applied alternative methods to diagnose the target disease for comparison. These methods included diagnosis according to a combination of tests (four studies), comparison to clinical follow-up (four studies), a pre-specified decision rule (one study), and a single gold standard applied only to a subgroup of patients (one study).

## Discussion

Our review on the use of panel diagnoses as reference standard in diagnostic studies reveals that panel diagnoses were mainly used in studies on psychiatric, cardiovascular, or respiratory conditions. Non-reporting of the panel methodology applied was frequent as 83% of all included studies did not report on all relevant items used in methods of the panel diagnosis necessary to replicate the study. The panel constitution and decision process differed substantially between studies, ranging from two to nine panel members, with large variations in the types of expertise represented in the panel. We found 17 different combinations of the three stages in the decision-making process as displayed in Table 7.

Complete and accurate reporting is a prerequisite for judging potential bias in a study and for allowing readers to apply the same study methods. In total, only 14 (17%) papers reported complete data on key issues such as the panel constitution, the information presented to the panel, and the exact decision process to determine the final diagnosis. This under- or even non-reporting shows that the standard of reporting of diagnostic studies should be improved. The STARD reporting guideline for diagnostic studies [10] does not include specific items on the use of panel diagnosis as reference standard. However, contrary to what one would expect, the completeness and thoroughness of reporting did not improve with time despite the publication of reporting guidelines in diagnostic research. Another problem we encountered in this review was unclear terminology. For example, the term “experts” was often used to describe the panel members. Yet little to no information was given to substantiate this claim, for instance by reporting on profession, expertise, or years of experience, and familiarity with the target disease or population of interest. Another ambiguous term was “consensus diagnosis.” It was often unclear whether the term consensus diagnosis was simply used as a synonym for panel diagnosis or whether it referred to a specific way of reaching agreement on the final diagnosis or target disease presence or absence among the panel members. Therefore, the term consensus diagnosis alone is not sufficient to describe the details of the reference standard. For example, instead of “the diagnosis was assigned in consensus,” it is more informative to describe the decision process as “the diagnosis was assigned in consensus after a group discussion.”

We used the key concept that reporting of research should enable replication. We therefore grouped items into four key domains: panel constitution, information presented to the panel, the decision process, and validity of the panel procedure. Using these four domains as guidance for reporting on the panel approach will aid replication of the study by others.

**Figure 3:**
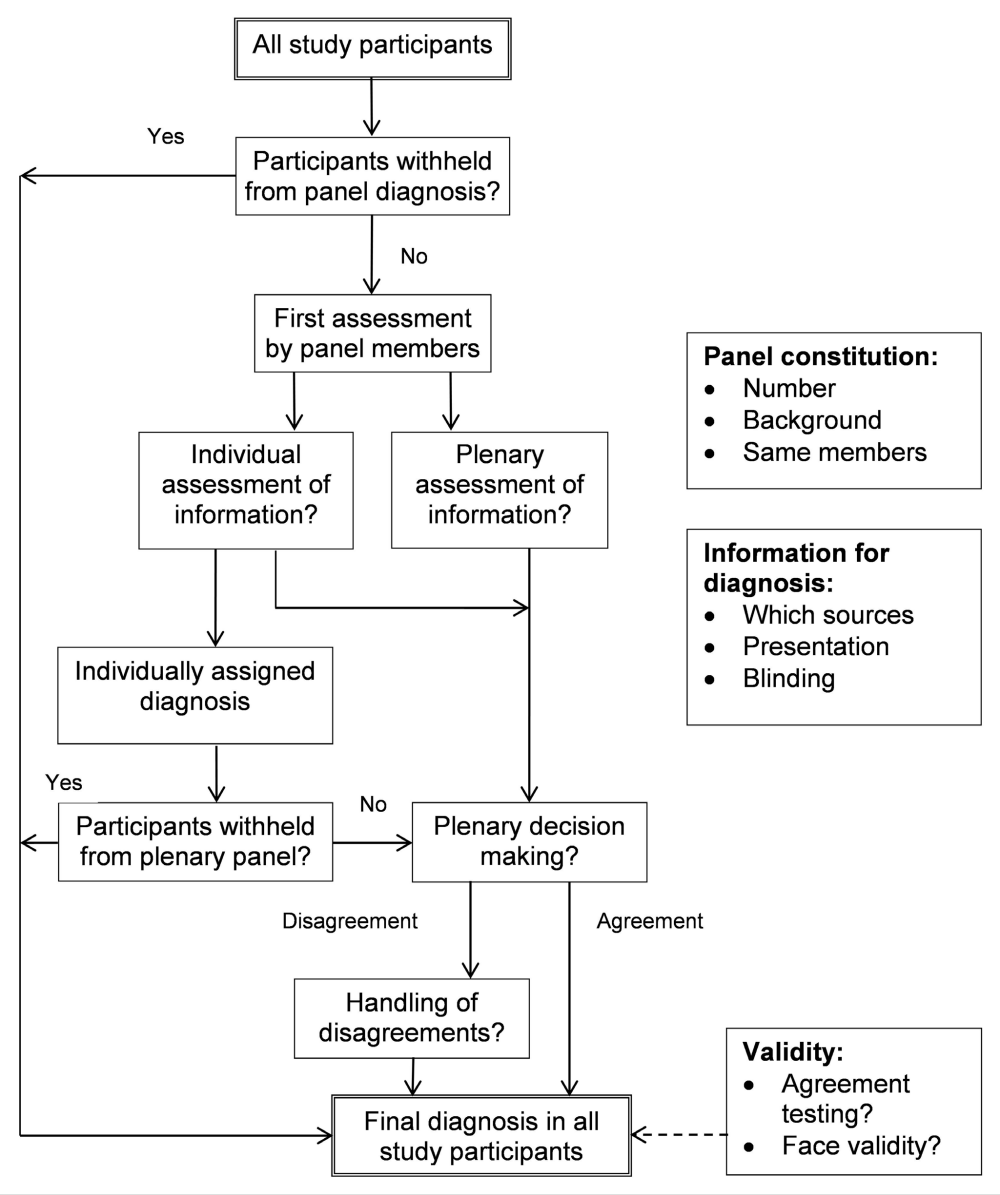
Flowchart of options to consider when planning and conducting panel diagnosis.

In Figure 3 and Table 8 we identify the various choices and decisions to be made before initiating a diagnostic study with panel diagnosis. We hope to encourage researchers to formally discuss these options when designing a new study rather than copying an approach from an existing study. Below, we discuss the options within each key domain based on the findings of our systematic review, supplemented by our experience (Figure 3; Table 8). We discuss these items in a cautious way as limited evidence or consensus exist on what should be considered preferred methodology for conducting a panel diagnosis. Further research into each of the decision we have identified is needed.

**Table 8:** Options to consider when reporting or designing a study using a panel diagnosis as reference standard.

**1**	**Panel constitution:**
	Number of members
	Odd number for voting
	Background of the members
	One or multiple areas of expertise represented?Broad or narrow expertise of the members?Years of experience
	Same panel constitution for all patients?
	Same member(s) present in every panel?Same expertise represented in each panel?
**2**	**Information presented to the panel:**
	Sources or domains of information
	e.g., history taking, physical examination, previous medical history, imaging, blood tests, follow-up, working diagnoses, etc.
	Information presented with or without interpretation?^a^
	Blinding?
	Blinding to what source of information?Complete or staged blinding?
**3**	**Decision process**
	Individual assessment of information by panel members BEFORE group meeting?
	Selected subgroups withheld from panel assessment?^b^
	Pre-specified decision rule?Agreement among members in individual assessment?
	Classification of the target condition
	Present/absent or multiple ordered categories?Probability estimations?
	Individual or plenary decision process?
	Handling of disagreements
	Plenary discussion?Additional expert and/or additional information?
**4**	**Validity of panel diagnosis**
	Agreement testing
	Reproducibility of plenary decision process?Inter-rater agreement?
	Face validity
	Comparing panel diagnosis to other possible reference tests:
	Comparison to clinical follow-up?Pre-specified decision rule?Obtain ‘gold standard’ in subgroup of patients?

Panel diagnosis definition: diagnosis based on multiple tests, agreed on by multiple experts.

a The default choice is paper-based summaries, including interpretation, of the information.

b The default choice is that all patients are assessed by the panel.

### Panel Constitution

Ideally, the same members should assess all patients to increase the reproducibility of the decision process. However, when this is not feasible, researchers can choose to have a particular member or a certain expertise to be present in each panel to help maintain a certain level of consistency. When voting is part of the decision process, an odd number of panel members should be considered. In the vast majority of studies, the panel consisted of three or fewer members, which seems low since the reason for using a panel diagnosis is that the final disease classification is not straightforward. Having more members is beneficial in avoiding incorrect decisions on the final diagnosis [93]. With the choice of panel members, one should consider whether all areas of expertise relevant to the target disease(s) are represented. While whether someone can be considered an expert is more or less subjective, reporting the area of expertise and the years of experience, as often done in inter-rater studies in imaging, provides useful information to the readers.

### Information Presented to the Panel

The information presented to the panel, as well as the format in which it is presented, is largely determined by the study aim and context. Researchers should provide the rationale for their choice of information used in the panel diagnosis, including references to existing guidelines, systematic reviews, and key papers on the diagnosis of the condition of interest. This will enhance the credibility (face validity) of their results.

A paper-based summary, containing the relevant patient information and test results, is considered the standard way of presenting. However, for certain tests, providing the “raw data,” such as 3D images in the case of complex bone fractures, should be considered. The credibility of final diagnosis can be improved by including follow-up information in the panel diagnosis. A drawback of including this information is a higher chance of missing data on follow-up and heterogeneity in additional diagnostic tests during follow-up, which will often not be random and may introduce verification bias [94].

### Decision Process

A disease can be classified as present or absent or can be rated using ordered categories to represent severity or certainty of diagnosis. Recording additional information on the certainty of the final diagnosis enables the researchers to perform additional analyses on the robustness of findings. Subsequent analysis could take the certainty of the final diagnosis into account, for instance by performing a weighted analysis.

The decision process itself is complex and several choices have to be made. The most commonly used options for this process are visualized in Figure S1. Individual assessment can be used to allow the panel members to read the information alone and make a preliminary diagnosis before discussion with other panel members. Also, this individual assessment can be used to define subgroups of patients that do not require evaluation by the entire panel, such as those who receive the same preliminary diagnosis from all panel members. Withholding these participants from the plenary discussions decreases the total workload for the panel members. Such subgroups can also be identified through application of a pre-defined decision rule. For example, a pre-defined combination of test results can clearly rule in or rule out disease in some patients, while the other patients need panel evaluation to determine the final diagnosis. In the plenary process, members influence each other which can either be beneficial or harmful [93]. Finally, the proportion of cases of disagreements should be reported, and the way the panel resolved the disagreement. More research is needed to determine if a plenary decision process is superior to an individual process, or vice versa. Procedures for resolving remaining disagreements are needed and should be formally decided upon at the beginning of the study.

### Validity of Panel Diagnosis

Although not frequently performed, the reproducibility of a panel diagnosis is easy to assess. Inter-rater agreement can be calculated in studies with individual assessment results. For the plenary decision process, reproducibility can be determined by reassessing a sample of the patients (obviously with the panel remaining blinded to their first judgment) and comparing the agreement. By comparing the panel diagnosis to clinical follow-up or another reference standard, insights in the validity of the panel diagnosis can be gained.

One of the authors of the included papers [62] stated that “it must be recognized that such diagnostic strategy may not be optimal. Expert opinion can be subjective and erroneous; this could lead to an overestimation or underestimation of the validity of all diagnostic methods in this study.” However, in the absence of a single gold reference test, panel diagnosis is a respected method to provide a solution. In a panel diagnosis, the tests are evaluated by multiple clinicians, and previous literature suggests that test evaluation by multiple clinicians leads to more accurate interpretation of index test results than evaluation by a single clinician [95],[96], accordingly suggesting that panel diagnosis is an acceptable method for diagnosis when a single gold standard is lacking [1],[6]. One of the included papers [71] reported “a great strength of the current study was its use of a structured consensus panel to determine a reference standard for each subject, without relying on a single test treated as the gold standard.” An advantage of panel diagnosis as opposed to composite reference standard or latent class analyses is the flexibility in the interpretation of the test results; each test result is interpreted in the context of all other information. This closely resembles clinical practice and therefore could lead to clinically relevant diagnoses [6],[7].

However, the use of panel diagnosis as reference standard also has disadvantages. The panel diagnosis approach is time and labor intensive. Also, the process is inherently more subjective and therefore results might be less reproducible than for other methods to deal with imperfect reference standards such as composite reference standard or latent class analyses. To quantify this problem, researchers could test the reproducibility of the decision process between panel members and across patients as a measure of the actual subjectivity of the panel diagnosis in the study.

Incorporation bias can be a serious threat to diagnostic studies. It refers to the situation where the results of the diagnostic tests under study (index test) are formally used when making the final diagnosis [6]. In cases of a panel diagnosis this occurs when the results of the test under study are part of the information available to the experts making the consensus diagnosis. The danger is that the results of the tests under evaluation receive too much weight in the decision-making process, leading to an overestimation of the accuracy of that test [6],[97],[98]. However, avoiding incorporation bias by withholding the index test results may in itself increase the risk of misclassification. One way to document the impact of the index test is to use staged unblinding in which the panel first classifies the disease status on the basis of all relevant information except the test under evaluation and again after revealing the index test results [6].

Alternative methods to deal with the absence of a single gold standard are composite reference standard [3] or latent class analyses [4],[5]. In composite reference standard, multiple test results are combined according to a pre-specified algorithm to rule the target disease in or out. These decision rules provide, like panel diagnoses, clinically interpretable diagnoses, but unlike the panel, the decision process is transparent and the same for all patients. Downsides of such decision rule is the limited number and types of tests that can be incorporated for decision making. Latent class analysis is a statistical method in which the probability of the disease status is modeled on the basis of the index tests and information available. However, the results are difficult to interpret clinically as the disease state is expressed in probabilities, rather than in a dichotomized (present or absent) fashion [4].

To our knowledge, this is the first systematic review on the methods applied in diagnostic studies using a panel diagnosis as the reference standard. Identification of studies using panel diagnosis through electronic searching was probably hampered by the fact that not all studies using this method report having done so in the abstract. Therefore, it is likely that we missed some studies. This, however, is unlikely to have had a meaningful impact on our findings about incomplete reporting and the variation present in the methodology of panel diagnoses. We have likely missed some additional papers because we have only searched a single electronic database (PubMed). However, we believe that completeness of the search was not the major issue for answering our research question, because the focus of our paper is on the method of panel diagnosis. To address this methodological issue, a comprehensive set of papers is likely to contain the relevant variations of the methodology of interest. This is very different from systematic reviews about the effectiveness of interventions, where the main aim is to validly estimate the weighted mean from all available studies in literature. A more extensive search might have identified some additional papers, but is unlikely to add relevant variations in the methodology already represented in the initial search. This phenomenon is known as theoretical saturation [9]. Moreover, each study identified within our search was carefully examined for the methods used in the panel diagnosis approach and the quality of reporting on these methods. As a result, a thorough search of Medline—the largest database of medical papers—will likely identify a sufficient number of papers reflecting all methods applied in panel diagnosis.

In conclusion, an expert panel diagnosis may be applied in diagnostic studies when a single gold reference standard is absent or not feasible and its use appears to be increasing in the medical literature. Our review revealed a large variation in applied methods as well as major deficiencies in the reporting of key features of the panel diagnosis process. To improve awareness about possible options when designing a diagnostic study with a panel diagnosis and how to report such studies, we provided some initial guidance highlighting key options in the methodology of panel diagnosis. The results of our review may serve as a starting point in the development of formal guidelines on methodology and reporting of panel diagnosis.

## Supporting Information

Figure S1 **Flowchart of the possible methods for decision making by panel diagnosis.**
(TIF)

Protocol S1 **Data extraction form.**
(DOCX)

Text S1 **PRISMA statement.**
(DOC)
