# Supplementary figures and images for: Use of Expert Panels to Define the Reference Standard in Diagnostic Research: A Systematic Review of Published Methods and Reporting
Source: PLoS Med. 2013 Oct 15;10(10):e1001531. doi: 10.1371/journal.pmed.1001531 (PMC3797139; doi:10.1371/journal.pmed.1001531)

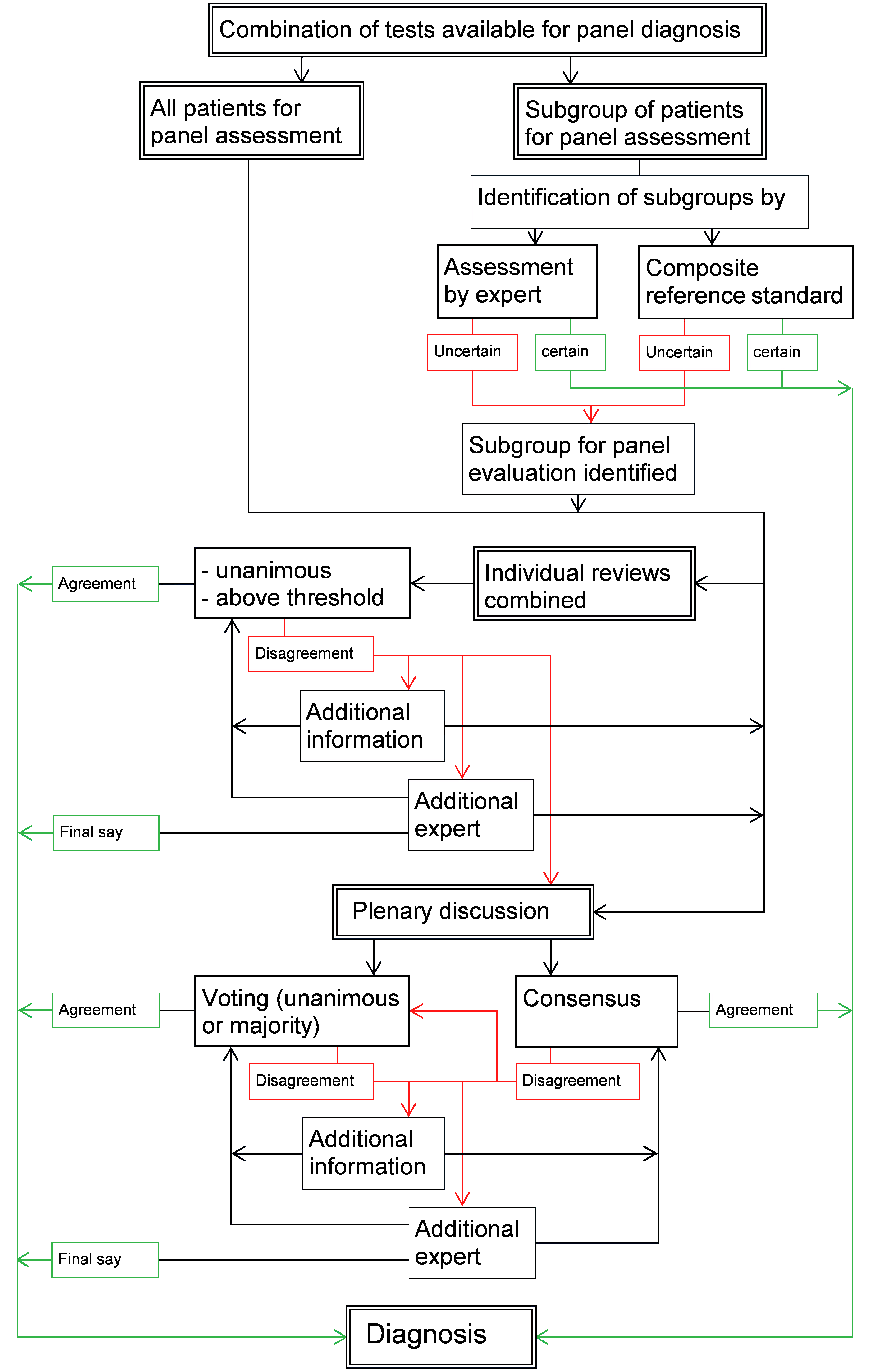

Supplement: Figure S1 — Flowchart of the possible methods for decision making by panel diagnosis. (TIF) [file pmed.1001531.s001.tif]
