# Supplementary material for: Use of Expert Panels to Define the Reference Standard in Diagnostic Research: A Systematic Review of Published Methods and Reporting
Source: PLoS Med. 2013 Oct 15;10(10):e1001531. doi: 10.1371/journal.pmed.1001531 (PMC3797139; doi:10.1371/journal.pmed.1001531)
Supplement: Protocol S1 — Data extraction form. (DOCX) [file pmed.1001531.s002.docx]

| **SOP overview expert diagnosis as reference test in diagnostic research** | | | |
| --- | --- | --- | --- |
|  |  | answers | description |
|  | **Administrative** | | |
| 1 | Paper ID (RM ID) |  | ID in Reference manager |
| 2 | Date of extraction |  | date of entry |
| 3 | Reviewer | LCMB, BDLB, JBR |  |
| 4 | Journal |  |  |
| 5 | First Author |  |  |
| 6 | Publication Year |  |  |
| 7a | Paper should be excluded | yes, no | If paper should be excluded from review, then also explain why (7b) |
| 7b | Why? |  | e.g. does not meet inclusion criteria (note which criterion), duplicate (note RM ID of original study), letter, editorial, case-report, commentary, review, etc. |
|  | **Research aim** | | |
| 8a | Type of main research question | Single test design, comparative design, add-on (added value), multivariable prediction, prevalence, technical paper, other (specify in 8b), unclear | Select the type of main research of the paper based on your thoughts. **If multiple**: select primary type of research question and note other(s) in 8b. (Single test: sens/spec/npv/ppv; comparative: kappa, measures of concordance; add-on: added value; multivariable prediction: development of model, multiple variables; prevalence; technical paper: on methods of consensus diagnosis) |
| 8b | Specify other |  |  |
|  | **Gold standard** | | |
| 10a | Reported reason why gold standard is not used | Not existing/high error, not ethical, index better than ref, gold standard too costly, other (specify in 10b), not reported | Reported reason why the gold standard is not used. **If multiple**: select primary reason note other(s) in 10b. |
| 10b | Specify other |  |  |
| 11a | Additional strategy used next to expert diagnosis to "check" final diagnosis? | yes, no | Is there additional evidence is used next to expert diagnosis? if not reported: select no If yes denote what info is used in 11b |
| 11b | What? |  | If 11a is yes, then say which type of extra info is used |
|  | **Experts** | | |
| 12a | Number of experts |  | The number of experts used for diagnosis. |
| 12b | Field of expertise | e.g. cardiology, pulmonology, psychiatrics, etc. | Specify the field(s) of expertise of the participating experts |
| 13a | Same members (persons) throughout the study? | yes, no, not reported | Are the same members used for the panel throughout the whole study? If no, specify in 13b t/m 13d |
|  | **In case of different panels (different members in panels):** | |  |
| 13b | Same expertise present in every panel? | yes, no, not all expertise (specify), not reported | Are the same expertises present in all panels? If "not all expertises", specify which expertise is present in all |
| 13c | How many different panel(s) were used? | <number>, not reported | If 13a is no, specify how many different panels were used |
| 13d | Was there at least one and the same person present in all panels? | yes (number), no, not reported | If 13a is no, specify how many member(s) were present in all panels |
|  | **Expert statements** | | |
| 14 | Number of target diseases |  | The number of target diseases of interest (according to the authors). Only count those that are used in analyses, otherwise they count as other diseases, not target diseases. **Specify in 15** |
| 15a | Target disease |  | If multiple target diseases: specify those of interest according to the authors. **Add ICD-10 code** |
| 15b | Target disease |  |  |
| 15c | Target disease |  |  |
| 16a | Statements on target disease(s) | present/absent; ordered categories (number), not reported | Specify the statements of the experts concerning the target disease(s) Ordered categories: for example no/possible/probable/yes, **note number of categories used** |
| 16b | Alternative diagnoses | yes (number), no, not reported | Specify the statements of the experts concerning the other disease(s)). If yes, note number of diseases. |
| 16c | % or chance of diagnose determined by expert panel? | yes, no, not reported | Is the 'certainty' of the diagnosis quantified in numbers? (In percentages, chance, etc.) |
|  | **Procedure** | | |
| 17 | *N* patients assessed by experts |  | number of patients that are assessed by the experts for final diagnosis |
| 18 | Is individual initial assessment of the evidence required | yes, no, not reported | where/when was the initial assessment the available evidence |
| 19a | Decision making process | consensus panel meeting, majority ruling, other (specify in 19b), not reported | Specify the decision making process: only one possible |
| 19b | Specify other |  |  |
| 20a | Procedure in case of remaining disagreement | consensus followed by majority voting, majority voting followed by consensus, additional experts are asked, other (specify in 20b), not reported | Specify procedure when disagreement remains. |
| 20b | Specify other |  |  |
| 21a | Available information for expert panel | medical history, physical examination, blood tests, function test, imaging, follow-up, other (specify) | classify which types of information is available to the expert panel (multiple answers possible) |
| 21b | Other than paper-based information available? | yes (specify), no, not reported | e.g. video, original images (without report/conclusions), audio, etc. |
| 21c | Staged consensus procedure? | yes (specify), no, not reported | example: stage 1 with information from history and physical examination, stage 2 with information from stage 1 and blood test |
| 22a | Blinding of panel to test(s)? | yes, no, not reported | Are the experts blinded to the index test? If no, describe in 19b how (and if) they deal with incorporation bias. |
| 22b | Blinded to which tests, why and how |  | Panel was blinded to which test(s)? how (staged consensus procedure, tests excluded from information for panel, etc.) |
| 23a | Reproducibility of consensus procedure tested | yes, no | Is the reproducibility of the expert diagnosis tested? If yes, specify statistical measure and proportion of cases used in 20b |
| 23b | Details of reproducibility |  | If 20a is yes, than specify: outcome measure, % retest, timeline (time between initial consensus diagnosis and retest diagnosis), etc. **Also extract total agreement and kappa (if possible)** |
|  | **Reporting panel/consensus** | | |
| 24 | Used (theoretical) references |  | Used references concerning expert diagnosis (theoretical and empirical references) |
| 25 | Limitations/strengths of panel diagnosis according to authors |  | Specify strengths and limitations of the used expert diagnosis mentioned by the authors |
| 26 | Remarks |  | Specify any remarks on the study and panel procedures |
